# Whole-Genome Sequencing Identifies a Novel Variation of *WAS* Gene Coordinating With Heterozygous Germline Mutation of *APC* to Enhance Hepatoblastoma Oncogenesis

**DOI:** 10.3389/fgene.2018.00668

**Published:** 2018-12-19

**Authors:** Li Zhang, Yaqiong Jin, Kai Zheng, Huanmin Wang, Shen Yang, Chenkai Lv, Wei Han, Yongbo Yu, Yeran Yang, Di Geng, Hui Yang, Tieliu Shi, Yongli Guo, Xin Ni

**Affiliations:** ^1^Center for Bioinformatics and Computational Biology, and the Institute of Biomedical Sciences, School of Life Sciences, East China Normal University, Shanghai, China; ^2^Beijing Key Laboratory for Pediatric Diseases of Otolaryngology, Head and Neck Surgery, MOE Key Laboratory of Major Diseases in Children, Pediatric Research Institute, Beijing Children’s Hospital, Capital Medical University, National Center for Children’s Health, Beijing, China; ^3^Biobank for Clinical Data and Samples in Pediatrics, Pediatric Research Institute, Beijing Children’s Hospital, Capital Medical University, National Center for Children’s Health, Beijing, China; ^4^Department of General Surgery, Wuhan Children’s Hospital, Wuhan, China; ^5^Department of Surgery, Beijing Children’s Hospital, Capital Medical University, National Center for Children’s Health, Beijing, China; ^6^Department of Otolaryngology, Head and Neck Surgery, Beijing Children’s Hospital, Capital Medical University, National Center for Children’s Health, Beijing, China

**Keywords:** hepatoblastoma, whole-genome sequencing, Wiskott–Aldrich syndrome (*WAS*), adenomatous polyposis coli (*APC*), cancer predisposition gene

## Abstract

Hepatoblastoma (HB), a leading primary hepatic malignancy in children, originates from primitive hepatic stem cells. This study aimed to uncover the genetic variants that are responsible for HB oncogenesis. One family, which includes the healthy parents, and two brothers affected by HB, was recruited. Whole-genome sequencing (WGS) of germline DNA from all the family members identified two maternal variants, located within APC gene and X-linked WAS gene, which were harbored by the two brothers. The mutation of APC (rs137854573, c.C1606T, p.R536X) could result in HB carcinogenesis by activating Wnt signaling. The WAS variant (c.G3T, p.M1-P5del) could promote HB cell proliferation and inhibit T-cell-based immunity by activating PLK1 signaling and inactivating TCR signaling. Further analysis reflected that WAS deficiency might affect the antitumor activity of natural killer and dendritic cells. In summary, the obtained results imply that an APC mutant together with an X-linked WAS mutant, could lead to HB tumorigenesis by activating Wnt and PLK1 signaling, inhibiting TCR signaling, and reducing the antitumor activity of natural killer and dendritic cells.

## Introduction

Although rare, hepatoblastoma (HB) is the most common and highly malignant liver tumor, arising in children under the age of 3 years. Its incidence is estimated to be approximately 1.2–1.5 million children per year globally, comprising about 1% of all pediatric cancers (Bell et al., 2017). Although most HB is sporadic, it has been described in association with a variety of inherited cancer syndromes, (Arthur Zimmermann, 2011) such as Beckwith–Wiedemann syndrome (Curia et al., 2008) and familial adenomatous polyposis (FAP). FAP is an autosomal-dominant cancer predisposition syndrome caused by germline mutations in *APC*, which encodes an antagonist of the Wnt signaling pathway (Arthur Zimmermann, 2011). Although *APC* mutations occur in 90% or more of FAP patients, their lifetime risk for HB is only 1.6% (Jasperson et al., 1993). This means that, in addition to *APC* germline mutation, there may be other driver mutations in HB cases with a family history of FAP.

Intriguingly, it was reported that HB is 50% more common in males than in females (Arthur Zimmermann, 2011). We thus assumed that X-linked gene mutations may also contribute to HB with *APC* mutation. Partly coinciding with this assumption, somatic gains of Xp or Xq were found in HB, and germline mutations in the X chromosome in X-linked disorders, such as Simpson–Golabi–Behmel syndrome and *MECP2* duplication syndrome, were also found accompanying HB (Terracciano et al., 2003; Arthur Zimmermann, 2011). However, these gene mutations can lead to HB without *APC* mutation. Thus, there may be other driver mutations accompanying *APC* mutation in HB with or without FAP.

In this paper, we report a new genotype [*APC* mutation and Wiskott–Aldrich syndrome (*WAS*) mutation] in two HB brothers with no obvious *WAS*-related disorder, which was discovered by applying WGS technology. *APC* rs137854573 c.C1606T (p.R536X) truncating mutation was previously reported to be related to colorectal cancer, while the novel *WAS* mutation (chrX:48,542,245, c.G3T) was identified as a new pathogenic variant in HB, leading to truncation involving deletion of the first 1–5 amino acids of the protein. These results provide new laboratory evidence for the clinical and prenatal diagnosis of HB; in particular, the new finding of an X-linked *WAS* mutation supports the notion that germline mutations in the X chromosome could be related to the pathogenesis of HB. Furthermore, the X-linked *WAS* mutation accompanying the *APC* mutation may explain the absence of HB in the mother but with both of the children suffering from HB.

## Materials and Methods

### Ethics Statement

The present study was approved by the Ethics Committee of Wuhan Children’s Hospital, and was conducted in accordance with the principles expressed in the Declaration of Helsinki. Participants and/or their legal guardians involved in this study provided written informed consent prior to inclusion in the study. Participants and/or their legal guardians also gave their written informed consent for the material to appear in Frontiers in Genetics and associated publications without limit on the duration of publication.

### Patients

**FIGURE 1:**
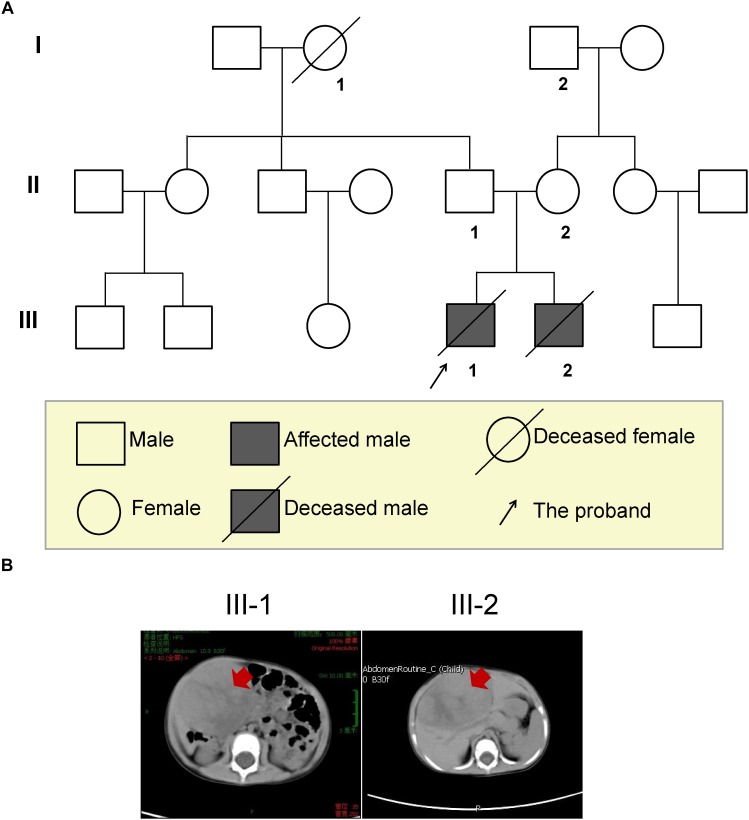
The three-generation pedigree of the family and imaging diagnosis of the HB brothers. **(A)** Solid symbols (squares = males, circles = females) indicate clinically affected individuals and open symbols indicate unaffected individuals. **(B)** Abdominal CT scan of both brothers confirmed the presence of the mass (red arrows), and measuring approximately 6.2 × 6.2 × 5.5 cm in the elder brother and 7.7 × 7.2 × 5.1 cm in younger brother.

This study included two young male patients who were brothers (III-1 and III-2) and the unaffected parents, as shown in Figure 1A. Genomic DNA samples were obtained with written informed consent. QIAamp^®^ DNA Blood Mini Kit (QIAGEN) was used for extracting genomic DNA from blood samples. DNA concentration was measured by Qubit^®^ DNA Assay Kit in Qubit^®^ 2.0 Flurometer (Life Technologies, Carlsbad, CA, United States).

### Whole Genome Sequencing

A total amount of 0.5 μg DNA per sample was used as input material for the DNA library preparations. Genomic libraries were prepared using the Illumina Truseq Nano DNA HT Sample Prep Kit following the manufacturer’s instructions. Libraries were analyzed for size distribution by Agilent 2100 Bioanalyzer. The clustering of the index-coded samples was performed on a cBot Cluster Generation System using Hiseq X PE Cluster Kit V2.5 (Illumina, San Diego, CA, United States) according to the manufacturer’s instructions. Then, the DNA libraries were sequenced on Illumina Hiseq platform and 150 bp paired-end reads were generated.

### Read Mapping and Variant Calling

Reads after quality control were aligned to the UCSC human reference genome (GRCh37/hg19 assembly) using BWA 0.7.12-r1039 mem mode (Li and Durbin, 2009). Samtools-0.1.18 was used for sorting, removing PCR duplicates, and building an index for the bam files. Variants were called using both the VarScan (Koboldt et al., 2012) (version 2.3.9) trio pipeline and the GATK (3.7-0) variant calling pipeline (McKenna et al., 2010). Variants jointly detected by both pipelines were retained for further analysis.

### Variant Annotation and Prioritization

The resulting variants were annotated and prioritized by ANNOVAR (Wang et al., 2010). A threshold of minor allele frequency (MAF < 0.01) from the 1000 Genomes Project Asian (Genomes Project et al., 2015) and ExAC (Lek et al., 2016) non-TCGA cohorts was used to screen rare variants. The pathogenicity of rare missense variants was evaluated by SIFT (Kumar et al., 2009), PolyPhen2 (Adzhubei et al., 2013), MutationTaster (Schwarz et al., 2014), and M-CAP (Jagadeesh et al., 2016). Other rare variants, including frameshift, prematurely truncating, and initial codon variants, were also retained for further analysis.

### Amplification and Sanger Sequencing of Mutation Sites

We amplified the two mutations in *WAS* and *APC* with a Veriti 96-well Thermal Cycler (Applied Biosystems, Thermo Fisher Scientific). The primer sequences used were as follows: *APC* (rs137854573) F: 5′-GTG ATA GGA TTA CAG GCG TGA GT-3′, R: 5′-TTA ACT TCT AAA GCA CAT TCC ATC A-3′; and *WAS* (chrX: 48542245) F: 5′-ACT TGT TTC CCT TGT CCC TTG T-3′, R: 5′-GAG TCG CTG GTT CTC GTG GT-3′. Mutations were confirmed by Sanger sequencing.

### Sources of Gene Expression Data

The normalized gene expression profiles for *WAS*-deficient and an HB patient with *APC* mutation were obtained from the ArrayExpress (Brazma et al., 2003) database with accession number E-MEXP-2282 (Mani et al., 2009) and from the Gene Expression Omnibus (GEO) database with accession number GSE75271 (Sumazin et al., 2017), respectively.

### Differential Expression Analysis and Gene Set Enrichment Analysis

As the sample size is small, we used log2 fold change to evaluate the differential expression level. Gene set enrichment analysis was implemented in GSEA (Subramanian et al., 2005) Desktop v2.2.4 with GseaPreranked mode. The gene sets were ranked by log2-transformed fold-change. Canonical pathways curated by MSigDB (Liberzon et al., 2011) (c2.cp.v6.0) were specified as the gene set database for enrichment analysis.

## Results

### Clinical Findings

A 15-month-old Chinese boy (the proband, elder brother, III-1; Figure 1A) was sent to the department of general surgery in Wuhan Children’s Hospital because of slight bulkiness of the abdomen without pain. Physical examination revealed an upper-right abdominal mass. Abdominal CT was performed and confirmed the presence of the mass, located in the inferior border of the right lobe (involving segments 5 and 6) of the liver and measuring approximately 6.2 × 6.2 × 5.5 cm (Table 1 and Figure 1B left). Alpha-fetoprotein (AFP) was significantly elevated, up to 1,000 IU/mL (normal < 5 IU/mL; Table 1). Following the COG (Children’s Oncology Group P9645) (Ortega et al., 2000) treatment protocol, the proband was treated with chemotherapy before attempted resection of the primary tumor. After surgical treatment for HB, pathological examination revealed hybrid fetal- and embryonic-type HB. In accordance with the International Children’s Liver Tumor Strategy Group (SIOPEL), the proband was classified into stage I of the PRE-Treatment Extent of tumor (PRETEXT) grouping system (children with stage I hepatocellular carcinoma have a better outcome) and as having low-risk HB. The proband was treated with 8 cycles of CDDP (cisplatin) plus ADR (Adriamycin) chemotherapy, which presented complete response (Supplementary Table S1). However, 43 months after diagnosis, this boy died of recurrent HB (Supplementary Table S1).

**Table 1:** Clinical features of the two hepatoblastoma patients.

	Patient 1 (III-1, Older)	Patient 2 (III-2, Younger)
Gender	Male	Male
Age at diagnosis (months)	15	12
Tumor stage^a^	I	III
Tumor size (cm)	6.2 × 6.2 × 5.5	7.7 × 7.2 × 5.1
Metastasis	No	No
Risk stratification	Low risk	Average risk
SIOPEL classification	Hybrid fetal and embryonic type	Hybrid fetal and embryonic type
HBV markers	HBsAb positive	HBsAb positive
AFP (IU/mL)	1000	3439
Prognosis	Deceased	Deceased


*^*a*^According to the PRE-Treatment Extent of tumor (PRETEXT) grouping system.*

The second case was the proband’s younger brother (14 months younger than his brother, III-2, Figure 1A), with similar clinical presentations. He was 12 months old at the time of diagnosis, and was also found to have bulkiness in the upper-right abdomen. An abdominal ultrasound was performed and revealed irregular echogenicity in the hepatic parenchyma. After hospitalization, a large liver mass of 7.7 × 7.2 × 5.1 cm in size was confirmed by abdominal CT (Table 1 and Figure 1B right). AFP was found to be extremely high, at 3,439 IU/mL (Table 1). The patient was also treated in accordance with the COG P9645 protocol (Malogolowkin et al., 2006). Pathological examination also disclosed hybrid fetal- and embryonic-type HB. This patient was diagnosed with PRETEXT stage III (Table 1) and an average risk of HB, in a poorer state than his elder brother. The younger brother was treated with 2 cycles of C5VD (cisplatin, 5-fluorouracil, vincristine and ADR) chemotherapy with stable disease (Supplementary Table S2). Then, ICE (iphosphamide, carboplatin, and etoposide) scheme and transcatheter arterial embolization (TAE) was used but still appeared stable disease (Supplementary Table S2). In 2017, he died of recurrent HB at the age of 4 years old.

The information of three generations of this family was collected (Figure 1A). The parents reported a normal pregnancy and delivery. Family history reveals no cancer or precancerous conditions. The grandmother (I-1) of the proband was died from traffic accident, and the grandfather (I-2) has congenital hand deformity. Other family members are healthy. Besides, the parents denied exposure to ionizing radiation, carcinogens, and virus infection, but accepted a healthy diet with plenty of fresh fruit and vegetables, and regular antenatal examination during the pregnancy period.

### Whole-Genome Sequencing Analysis

**FIGURE 2:**
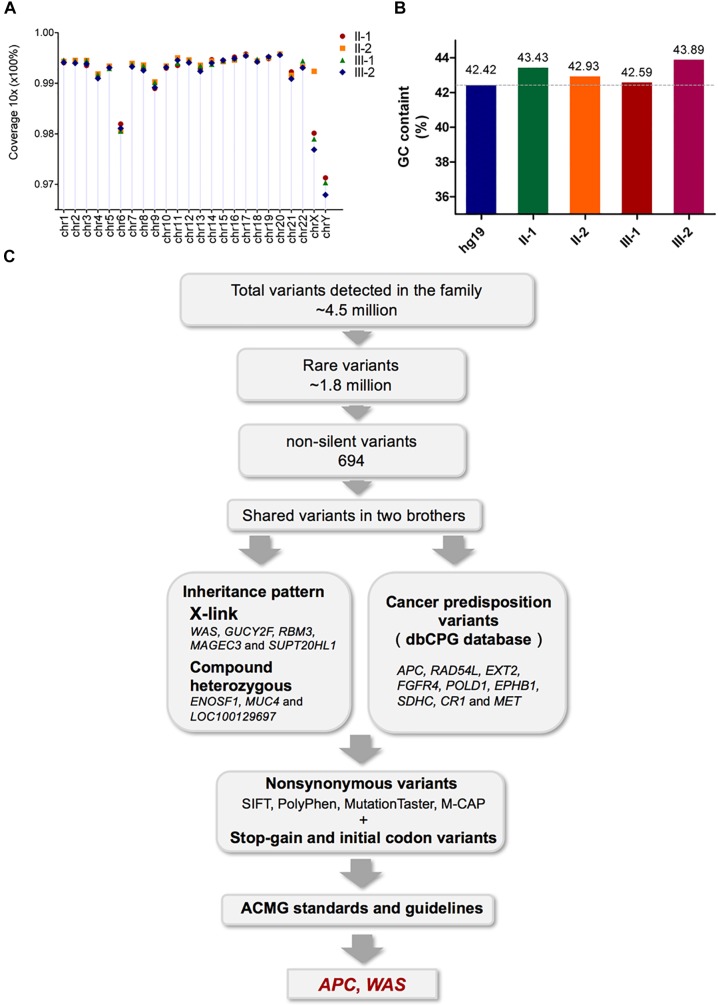
Assessment of the WGS data quality of the four family members and validation of the pathogenic mutations by Sanger sequencing. **(A)** The percentage of 10-fold coverage for each sample is arranged by chromosomes. Overall, the average coverage for each chromosome and sample was higher than 96%. **(B)** Comparison of the average GC content for the WGS reads with the human reference genome (hg19). The average GC content for each sample was around 43%, and no significant bias was observed. **(C)** Workflow for the identification of pathogenic mutations.

The fact that two brothers in the same family suffered from HB strongly suggested that their hepatocarcinogenesis was caused by germline variants. Therefore, to identify the causative variants contributing to the hepatocarcinogenesis, we performed high-coverage and high-quality WGS of all family members. Specifically, more than 96.5% of the whole genome had 10-fold coverage and showed high consistency among the experiments (Figure 2A). Bias in GC content was not observed in any of the experiments, which ranged from 42.59 to 43.89% (Figure 2B). In addition, more than 85% of sequenced bases reached Q_30_ based on the analysis of Phred-scaled quality score (Supplementary Table S3).

The variant calling analysis identified about 4.5 million variants in each family member (Supplementary Table S4). Of these, a total of 1.8 million rare variants were selected based on MAF < 1%. Functional annotation of the rare variants characterized 694 non-silent variants harbored by both patients, including 18 splicing variants, 17 frameshift insertions or deletions (indels), 645 non-synonymous variants, 13 stop–gain variants, and 1 stop–loss variant (Supplementary Table S4).

As shown in Figure 2C, pathogenicity analysis for further candidate mutations was performed (Jin et al., 2018). Nine variants in nine cancer predisposition genes (annotated by the dbCPG database; Wei et al., 2016), as well as variants satisfying inheritance patterns including five X-linked genes and eight compound heterozygous mutations in three genes, were observed (Supplementary Tables S5, S6). Meanwhile, no *de novo* germline mutations or autosomal recessive mutations were found in the non-silent variants shared by the two brothers. All non-synonymous variants were filtered using the pathogenic scores in SIFT (Kumar et al., 2009) (≤0.05), PolyPhen2 (Adzhubei et al., 2013) (≥0.957), MutationTaster (Schwarz et al., 2014) (‘disease causing’), and M-CAP (Jagadeesh et al., 2016) (>0.025), and only RAD54L(chr1:46725718 G > C, p p.L118F) was recognized (Supplementary Table S6). The pathogenicity of the stop–gain mutations, initial codon variants, and filtered non-synonymous variants was finally evaluated according to the American College of Medical Genetics and Genomics and the Association for Molecular Pathology (ACMG) standards and guidelines, (Richards et al., 2015) from which only two variants in the *APC* and *WAS* genes were identified (Table 2).

**Table 2:** Summary of functional variants in the brothers with hepatoblastoma.

Chr	Position	Ref	Alt	Variant	Effect^a^	Evidence^a^	Origin
Chr5	112164586	C	T	APC.p.R536X	Pathogenic(Ia)	PVS1, PS1, PP1-S, PM2, PP3, BP1	Maternal
ChrX	48542245	G	T	WAS.p.del1-5	Pathogenic(II)	PS1, PS3, PM2, PM4, PP3	Maternal
Chr1	46725718	G	C	RAD54L p.L118F	Not enough evidence	PM2, PP3, BP4, PM5	Paternal


*^*a*^According to the standards and guidelines for the interpretation of sequence variants of the American College of Medical Genetics and Genomics and the Association for Molecular Pathology.*

### Validating the *APC* Mutation in the HB-Affected Brothers and Determining Its Mechanistic Association With HB Accompanying Dysfunctional APC

**FIGURE 3:**
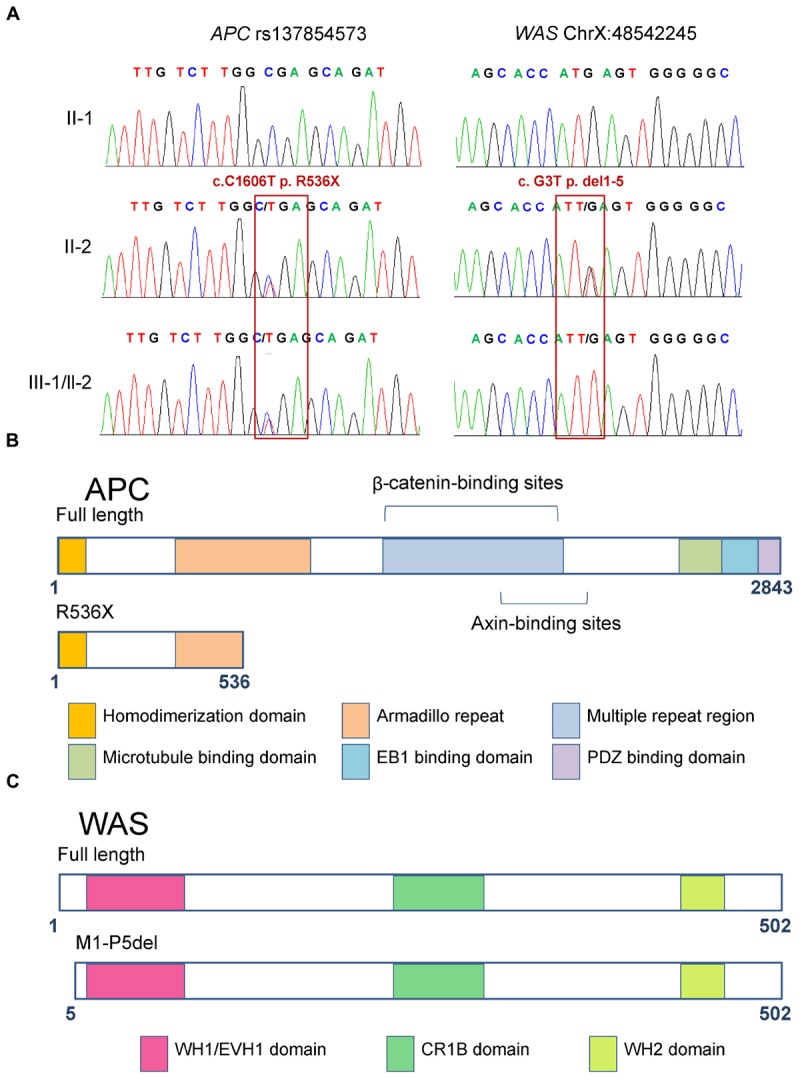
Functional domains of full-length and mutant APC and WAS proteins. **(A)** Sanger sequencing confirmed the heterozygous mutations rs137854573 (c.C1606T, p.R536X) of the *APC* gene and X-linked *WAS* mutation (c.G3T, p.M1-P5del). The two patients (III-1 and III-2) carried both of these variants, inherited from their mother. **(B)** The full-length APC protein harbors six functional domains, namely, homo-dimerization, armadillo repeat, multiple repeat region, microtubule binding, EB1 binding, and PDZ binding domains. The variant in APC results in premature truncation at the 536th amino acid of the APC protein, which only includes the homo-dimerization and incomplete armadillo repeat domains. **(C)** The full-length WAS protein contains domains including WH1/EVH1, CR1B, and WH2. The variant in WAS results in deletion of the first five amino acids, but does not abolish any of the functional domains.

According to the results above, the premature truncating variant in *APC* (rs137854573, c.C1606T, p.R536X; Figures 3A,B) was shown to have been inherited from their mother, which was validated by Sanger sequencing (Figure 3A). Based on the annotation from ClinVar and COSMIC, rs137854573 in *APC* has been reported to be a dominant variant causing FAP (Fodde et al., 1992), and detected in colon cancer and ovarian cancer (Forbes et al., 2016). Our findings revealed that rs137854573 in *APC* could be a variant with an important role in the pathogenesis in the brothers.

**FIGURE 4:**
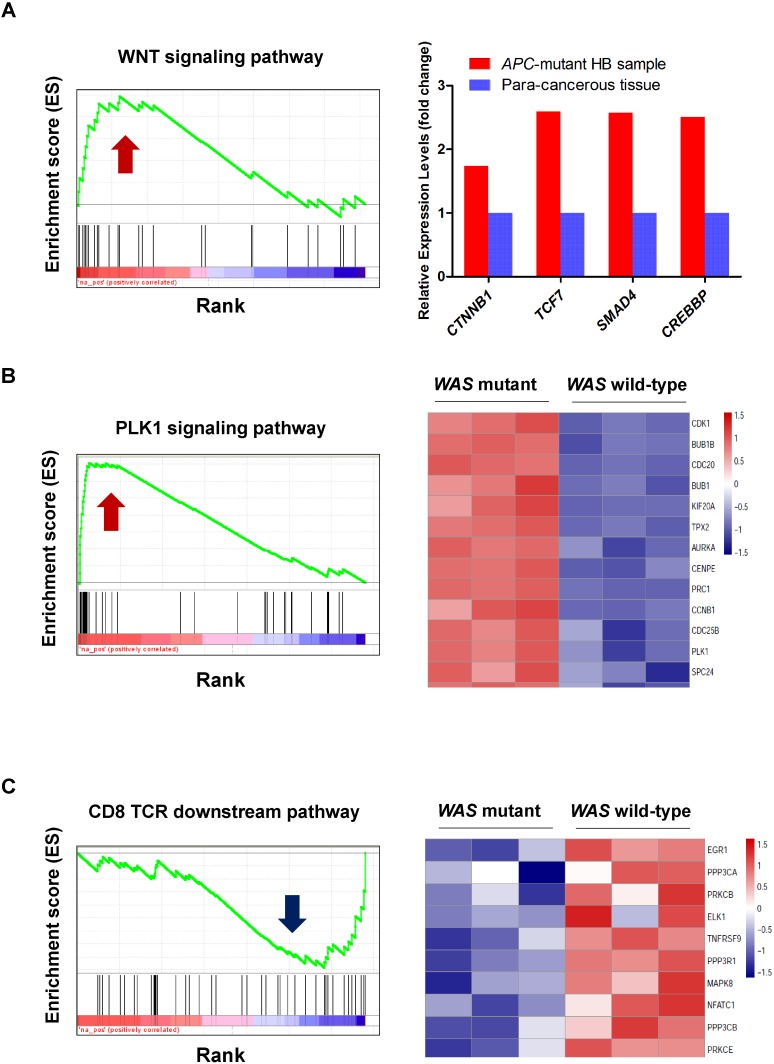
Gene set enrichment analysis (GSEA) revealed activated or inactivated genes and pathways induced by the loss of WAS or APC. **(A)** Upregulation of the WNT signaling pathway and its critical transcriptional regulators, CTNNB1, TCF7, SMAD4, and CREBBP, in an HB sample with APC mutation. The normalized expression profiles of the HB sample with APC mutation and para-cancerous tissues were obtained from the Gene Expression Omnibus (GEO, GSE75271). **(B,C)** Display the upregulation of PLK1 signaling pathway and downregulation of CD8-TCR downstream pathway in WAS mutant mice, respectively. The normalized expression profiles of wild-type and WAS mutant mice were obtained from the ArrayExpress database (E-MEXP-2282).

The loss of function in APC can result in the activation of Wnt signaling in colorectal cancer (Fodde et al., 2001) and hepatocellular carcinoma (Zhan et al., 2017). To determine whether Wnt signaling is activated in HB patients with inactivated APC, we compared the expression profile of the tumor tissue of an HB patient carrying a frameshift germline mutation in *APC* (p.L666X) with that of normal controls from the work of Sumazin et al. (2017). Gene set enrichment analysis (GSEA) showed that Wnt signaling was activated in this HB patient (Figure 4A, FDR < 0.01). Further analysis found that most Wnt substrates, including *CTNNB1*, *TCF7*, *SMAD4*, and *CREBBP*, were highly upregulated in APC-deficient cells (Figure 4A). This suggested that inactivated *APC* could also activate Wnt signaling in our patients. However, this finding also raised the question of why the mother of these brothers had not suffered from HB or another cancer.

### Validating the *WAS* Mutation and Determining Its Mechanistic Effect Using Public Data

In addition to premature truncating variants, initial codon variants were also considered as candidate pathogenic variants. We found a hemizygous variant in the initial codon of *WAS* (c.G3T, p.M1-P5del) in both patients, which resulted in a smaller WAS protein (Figures 3A,C). Notably, the same smaller WAS protein resulting from another variant in *WAS* (c.A1T, p.M1-P5del) was reported to alter the normal TCR-beta repertoire in a patient with WAS (Du et al., 2006). Wiskott–Aldrich syndrome patients have a higher than normal risk of developing malignancies. Our results indicated that this X-linked *WAS* (c.G3T, p.M1-P5del) mutation could be another key mutation that can promote the development of HB.

Wiskott–Aldrich syndrome deficiency is related to a higher risk of malignancies. To investigate the potential molecular mechanism behind WAS-deficiency-induced carcinogenesis, expression data from WAS-deficient bone marrow mononuclear cells and their controls were collected (Mani et al., 2009). GSEA revealed that loss of function in WAS could activate PLK1 signaling (FDR < 0.001, Figure 4B). Further analysis found that most PLK1 substrates, including *BUB1B*, *CCNB1*, *CDC25C*, *PRC1*, and *FBXO5*, were highly upregulated in WAS-deficient cells (Figure 4B). PLK1 is one of the most important serine/threonine-protein kinases involved in mitosis, which is overexpressed in human tumors (Strebhardt and Ullrich, 2006). We thus assumed that WAS-deficiency-induced PLK1 signaling transduction may contribute to the hepatocarcinogenesis in HB. GSEA also revealed that the loss of function in WAS inactivated the CD8-TCR downstream pathway (*P*-value < 0.05) (Figure 4C). Notably, NFATC1, a key regulator of T-cell development and function, (Macian, 2005) was downregulated in WAS-deficient cells, indicating that WAS deficiency could inactivate TCR signaling (Figure 4C). Based on these results, we proposed that the loss of function of WAS contributed to HB oncogenesis through activating PLK1 signaling and inhibiting the TCR signaling pathway.

**FIGURE 5:**
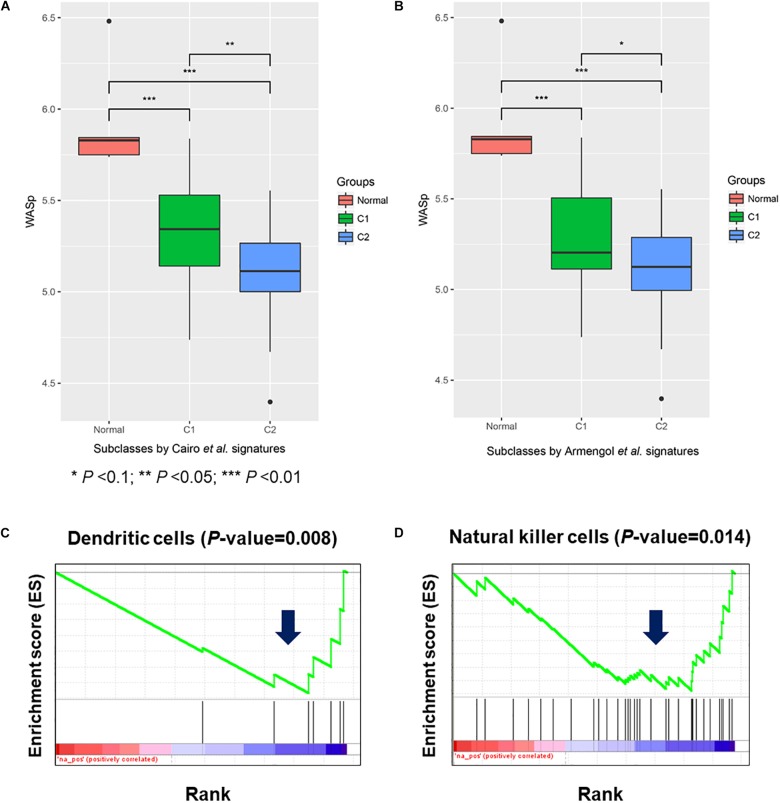
Wiskott–Aldrich syndrome expression in hepatoblastoma (HB) and potential impact of its inactivation on dendritic and natural killer cells. **(A,B)** The expression level of WAS in normal liver, HB C1, and C2 subclasses (GSE75285). The down-regulation of specifically expressed genes in dendritic **(C)** and natural killer cells **(D).**

To further demonstrate the role of WAS in HB oncogenesis, we investigated expression levels of *WAS* in HB and normal liver using the gene expression dataset from Sumazin et al. (2017), and observed WAS down-regulated in HB, as compared with normal controls (Figures 5A,B, one-side Wilcoxon rank-sum test, *P*-value < 0.05). Particularly, WAS was specifically down-regulated in C2, a subclass with poorer prognosis defined by Cairo et al. (2008) and Armengol et al. (2011) indicating that downregulation of *WAS* was closely associated with poor prognosis. In accordance with WAS protein expressed exclusively in hematopoietic cells, (Derry et al., 1994; Stewart et al., 1996; Parolini et al., 1997) Catucci et al. (2014) found that WAS deficiency in natural killer (NK) and dendritic cells (DC) could affect antitumor immunity. We thus performed GSEA on the differentially expressed genes in HB tissues, and revealed that the NK and DC cells-specifically expressed genes were significantly down-regulated in HB tissues as compared with normal controls (Figures 5C,D), indicating that lack of WAS could induce the hepatocarcinogenesis because of defective immune surveillance by NK and DC cells.

## Discussion

Next generation sequencing has been widely used to decipher the underlying variants for rare disease (Jia and Shi, 2017; Qi et al., 2017). Here, we report that WGS of germline DNA in two brothers with HB and their unaffected parents revealed two independent mutations in *APC* and *WAS*. Both brothers harbored these two variants, which were both transmitted from their mother. In addition, our results provide useful information to those couples, who have one or two children with malignant tumors and are generally eager to know the risk of such disease in the third child. Moreover, if the mother is pregnant with a third child, our research could provide pre-conception genetic result of the brothers, thus make the prenatal diagnosis more accurate.

### Loss of APC Function Leads to HB by Activating Wnt Signaling

To the best of our knowledge, no previous reports have been described an association between rs137854573 of APC and HB. However, our findings predicted that heterozygous rs137854573 would terminate translation at 536th amino acid, thus leading to a truncation associated with the lack of important domains of APC, including beta-catenin- and AXIN-binding sites (Figure 3B). Loss of APC function by this variant has been suggested to activate Wnt signaling in colorectal cancer and hepatocellular carcinoma, (Klaus and Birchmeier, 2008) but this is unknown in HB. By integrative analysis of HB genome and transcriptome data, activated Wnt signaling was found in HB tissue with mutant *APC*. This supports the notion that activation of the canonical Wnt-signaling pathway can be caused by germline alterations including *APC* mutations (Sumazin et al., 2017).

Hepatoblastoma is associated with FAP, (Hirschman et al., 2005) which is an autosomal-dominant cancer predisposition syndrome caused by germline mutations in *APC* (Rustgi, 2007). To demonstrate the pathogenicity of the *APC* truncating mutation, we systematically investigated the literatures. Firstly, three previous studies (Friedl and Aretz, 2005; Truta et al., 2005; Shirts et al., 2016) reported a total of 12 FAP cases caused by this *APC* mutation (rs137854573). Although no previous study reports the directly association between rs137854573 and HB, Rivera et al. (2011) has found that one FAP case with rs137854573 was diagnosed with osteoma, and another two cases of HB with two *APC*-truncating mutations (p.R1114X and p.Q1294X) had symptoms of FAP, suggesting that *APC*-truncating mutation carriers had a higher risk of comorbidity between FAP and cancers like osteoma and HB. Interestingly, the mother of the patients, also carrying the *APC* truncating mutation, has not exhibited any symptoms of FAP or colon cancer, but has a higher than normal risk of developing APC-related diseases, such as FAP and colorectal cancer. Notably, Yang et al. (2018) has reported four HBs caused by APC mutations, including two truncating mutations and two missense mutations, but without any family history, such as FAP, polyposis, or early colectomy. As such, it is essential to maintain regular follow-ups of the mother’s health.

### Loss of *WAS* Function Accompanying *APC* Mutation in Two Brothers With HB

Next-generation sequencing technology provides a perfect tool to investigate potential gene mutations that may have effects on the oncogenesis of HB. Our results indicate that maternal X-linked *WAS* (c.G3T, p.M1-P5del) mutation is another key mutation promoting the development of HB. It has reported that the smaller WAS p.M1-P5del has been discovered as a second-site mutation by Du et al. (2006), and is insufficient to reconstitute the normal TCR-beta repertoire (Du et al., 2006), which is in accordance with our result that *WAS* knockout affected the TCR signaling pathway. Furthermore, *WAS* pathogenic mutation could weaken the antitumor immunity by the immune surveillance of DC and NK cells. In addition to the negative regulation of TCR signaling, knockout of *WAS* could also activate PLK1 signaling, which has been suggested to serve as oncogenic signaling in most human cancers. We investigate the specific interplaying of these pathways, such as WNT, PLK, and TCR signaling pathways, affected by these two genes. However, there was no direct interaction between these pathways. Alternatively, we found that CDC42 directly interacted with both APC and WAS in the regulation of actin cytoskeleton (KEGG pathway). The three proteins, CDC42, APC, and WAS, jointly participated in the regulation of actin cytoskeleton. We then speculated that inactivation of both APC and WAS may change the activity of the regulation of actin cytoskeleton, thereby leading to tumorigenesis.

In addition to the mutation status, the methylation state of the two genes was also importantly related to tumor, which we did not consider in this study. Further study will focus on the consequences of methylation state of these potential driver genes on tumorigenesis and the interactions between methylation state and mutation status in familial pediatric cancer. Malignancies, such as lymphoma, spinalioma, seminoma, acute lymphoblastic leukemia, and pancreatic cancer, are frequently encountered in individuals with pathogenic variants of *WAS*. Our finding indicates that which in coordination with the promotion of Wnt signaling by the *APC* mutation, could have contributed to HB oncogenesis. Intriguingly, in TCGA data on rectal adenocarcinoma, we found that two males among 137 patients harbored *WAS* mutations (R309H and D292N), who also carried APC mutations (R1114X; S1400L/S2761L). This suggests that WAS mutations in combination with APC mutations may enhance tumorigenesis. The concurrent presence of the mutated forms of APC and WAS was not specific to HB, it has been reported that dysregulation of WAS or APC relates to the onset and progression of other cancers, like decreasing WAS expression in chronic myeloid leukemia (Pereira et al., 2017) and APC mutant in colorectal cancer. Our result raises an interesting question how co-presence of APC and WAS mutation could cause different malignancies.

### WAS-Related Disorder and X-linked Cancers

Pathogenic variants in *WAS* often cause *WAS*-related disorders, including Wiskott–Aldrich syndrome, X-linked thrombocytopenia, and X-linked congenital neutropenia (Chandra et al., 1993) which are a spectrum of disorders of hematopoietic cells. Surprisingly, our patients showed no WAS-related symptoms except malignancy. Most pathogenic variants of *WAS* lead to stop–gain or frameshift alterations of the WAS protein, and these truncations are associated with the loss of most WAS function. Du et al. (2006) found the smaller WAS (same truncation with our patient, p.M1-P5del) could rescue most function of NK and T cells, but is still not sufficient to reconstitute normal TCR-beta although it confers growth advantage over WAS negative T cells. This may explain the reason why our patients carry WAS mutant but don’t have a symptoms of WAS syndrome. In the present study, the X-linked *WAS* mutation may thus provide genomic evidence in supporting of HB having a tendency to occur in males (Arthur Zimmermann, 2011). Our discovery is not the first time that HB has been reported to be accompanied by an X-linked gene mutation. For example, it has been reported that HB occurred in single Simpson–Golabi–Behmel syndrome patients frequently carrying mutations in the glypican 3 gene (Xiong et al., 2015). In addition, Terracciano et al. (2003) found that over 77.4% (24/31) of HB tissues harbor X-chromosome gains. These findings together provide support for our hypothesis that X-linked mutations are associated with males with HB.

### Limitations

Our observations are limited by the fact that the mother who carries *APC* mutation has not shown any *APC*-related disorders. However, the mother still has a higher risk of developing APC-related diseases, such as FAP and colorectal cancer. Besides, the parents ruled out any family history of cancer and FAP, and unfortunately permission was not granted to obtain blood samples from relatives of the mother. This prevented the creation of a more extended pedigree for this family. For a similar reason, the HB tumor tissue of the brothers could not be obtained, which prevented determination of the precise mechanism of HB in the patients.

## Conclusion

Our data suggest that maternal heterozygous *APC* mutation (rs137854573, p.R536X) and a novel X-linked mutation in *WAS* (c.G3T, p.M1-P5del) may have cooperated to promote HB in the brothers. Our results also indicate the potential mechanisms by which these *APC* and *WAS* mutations may induce hepatocarcinogenesis, namely, through dysregulated Wnt, PLK1, and CD8-TCR signaling pathways, and reduced activity of NK and DC cells. Besides, our findings partially explain why the same *APC* mutant in some carriers leads to the development of FAP, while in others it is associated with HB. For further investigation of the genotype–phenotype relationship in HB, efforts should be made to recruit more families affected by this disease, despite their rarity.

## Author Contributions

LZ, YJ, and KZ: conducted the experiments, analyzed the data, and wrote the manuscript. HW: conducted the experiments, and wrote, reviewed, and edited the manuscript. SY, WH, YbY, YrY, DG, and HY: conducted the experiments. CL: analyzed the data. TS, YG, and XN: designed the study, wrote, reviewed, and edited the manuscript, and administrated the project.

## Conflict of Interest Statement

The authors declare that the research was conducted in the absence of any commercial or financial relationships that could be construed as a potential conflict of interest.
