# Supplementary material for: Whole-Genome Sequencing Identifies a Novel Variation of WAS Gene Coordinating With Heterozygous Germline Mutation of APC to Enhance Hepatoblastoma Oncogenesis
Source: Front Genet. 2018 Dec 19;9:668. doi: 10.3389/fgene.2018.00668 (PMC6305990; doi:10.3389/fgene.2018.00668)
Supplement: Supplementary file 1 [file Table_1.DOCX]

**Table S1 Patient 1 (elder brother) chemotherapy regimens and response**

| Therapy | AFP (IU/ml) | Primary tumor (cm) | Response to therapy |
| --- | --- | --- | --- |
| At diagnosis | 1000 | 6.2×6.2×5.5 |  |
| Surgery |  |  |  |
| Cycle 1: CDDP+ADR |  | Negative |  |
| Cycle 2: CDDP+ADR | 18.65 | Negative |  |
| Cycle 3: CDDP+ADR | 6.92 | Negative |  |
| Cycle 4: CDDP+ADR | 446.1 |  |  |
| Cycle 5: CDDP+ADR |  |  |  |
| Cycle 6: CDDP+ADR |  |  |  |
| Cycle 7: CDDP+ADR | 18.43 |  |  |
| Cycle 8: CDDP+ADR | 19.93 | Negative | Complete response (CR) |
|  |  |  |  |
| 43 months after diagnosis | 2.01 | Retroperitoneal soft tissue recurrence | Recurrence |
| Treatment withdrawal |  |  |  |
