# Supplementary material for: Whole-Genome Sequencing Identifies a Novel Variation of WAS Gene Coordinating With Heterozygous Germline Mutation of APC to Enhance Hepatoblastoma Oncogenesis
Source: Front Genet. 2018 Dec 19;9:668. doi: 10.3389/fgene.2018.00668 (PMC6305990; doi:10.3389/fgene.2018.00668)
Supplement: Supplementary file 2 [file Table_2.DOCX]

**Table S2 Patient 2 (Younger brother) chemotherapy regimens and response**

| Therapy | AFP (IU/ml) | Primary tumor (cm) | Response to therapy |
| --- | --- | --- | --- |
| At diagnosis (core needle biopsy) | 3439 | 7.7×5.1×7.2 |  |
| Cycle 1: C5VD |  |  |  |
| Cycle 2: C5VD | 765.5 | 8.3×6.5×8.1 | Stable disease (SD) |
| Cycle 3: ICE | 1180.0 | 8.4×6.3×5.5 |  |
| TAE | 446.1 | 7.6×7.7×6.4 | Stable disease (SD) |
| Treatment withdrawal |  |  |  |
