# Supplementary material for: Whole-Genome Sequencing Identifies a Novel Variation of WAS Gene Coordinating With Heterozygous Germline Mutation of APC to Enhance Hepatoblastoma Oncogenesis
Source: Front Genet. 2018 Dec 19;9:668. doi: 10.3389/fgene.2018.00668 (PMC6305990; doi:10.3389/fgene.2018.00668)
Supplement: Supplementary file 4 [file Table_4.DOCX]

**Table S4 Whole-genome sequencing statistics for the family**

| Family member | # of reads | # of mapped reads | # of variants | # rare variants (MAF<0.01) | Average DOC |
| --- | --- | --- | --- | --- | --- |
| II-1 | 1,662,996,306 | 1,658,691,911 | 4,416,230 | 1,383,758 | 82.93 |
| II-2 | 1,398,037,988 | 1,393,794,262 | 4,433,218 | 1,368,962 | 69.69 |
| III-1 | 1,574,057,632 | 1,569,039,574 | 4,396,807 | 1,365,821 | 78.45 |
| III-2 | 1,411,699,962 | 1,408,031,432 | 4,374,042 | 1,350,364 | 70.40 |

MAF, minor allele frequency

DOC, depth of coverage
